# IMPACT-CABG Trial: Implantation of CD133^+^ Stem Cells in Patients Undergoing Coronary Bypass Surgery—Presentation of the First Treated Patient

**DOI:** 10.1155/2011/685394

**Published:** 2011-06-22

**Authors:** Jessica Forcillo, Louis-Mathieu Stevens, Samer Mansour, Ignacio Prieto, Denis-Claude Roy, Nicolas Noiseux

**Affiliations:** ^1^Department of Cardiac Surgery, CHUM, 3840 Rue Saint-Urbain, Montréal, QC, Canada H2W 1T7; ^2^Surgery Department, Université de Montréal, Montréal, QC, Canada H3C 3J7; ^3^CHUM Research Center (CRCHUM), Montréal, QC, Canada H2W 1T7; ^4^Cardiology Department, CHUM, Montréal, QC, Canada H2W 1T7; ^5^Hematology Department, Maisonneuve-Rosemont Hospital, Montréal, QC, Canada H1T 2M4

## Abstract

The IMPACT-CABG study is the first Canadian randomized-controlled phase II clinical trial aiming to assess the effect of intramyocardial (IM) injections of CD133^+^-selected stem cells in patients referred for coronary artery bypass graft (CABG) with a “chronic” myocardial infarction and persistent left ventricular dysfunction. Patients are followed for 2 years with different imaging techniques including the stress magnetic resonance to evaluate the global and regional myocardial viability. Before the beginning of the randomization, the 5 first patients are treated in an open-label fashion to assess safety and feasibility of the IM CD133^+^ injections. Herein, we report the first Canadian patient treated with IM injection of CD133^+^ cells during CABG surgery as part of the IMPACT-CABG trial.

## 1. Introduction

The IMPACT-CABG study is the first Canadian randomized-controlled phase II clinical trial testing the effect of intramyocardial- (IM-) selected autologous CD133^+^ bone marrow (BM) stem cells as compared to placebo in patients referred for coronary artery bypass graft (CABG) surgery with chronic myocardial infarction (MI) and left ventricular (LV) dysfunction. However, before the beginning of the randomization and to test the safety and feasibility of the CD133^+^ cells collection and IM injection, the first five patients are treated in an open-label fashion. Herein, we report the first Canadian patient treated with CD133^+^ cells during CABG.

## 2. Case Report

**Figure 1:**
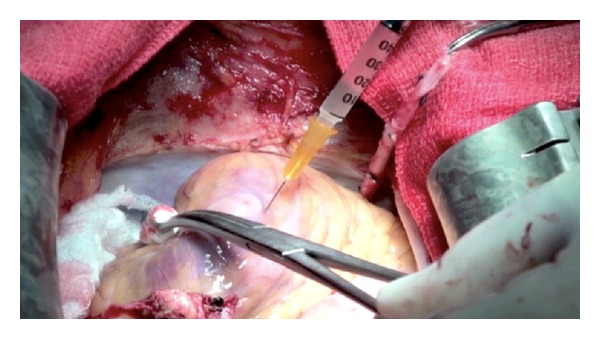
Picture showing the intraoperative injection of the CD133^+^ stem cells into the infarcted area and infarct border zone.

A 59-year-old male known for previous smoking, hypertension, insulin-treated type II diabetes, and a mild chronic renal insufficiency (estimated glomerular filtration rate of 47 mL/min/1.73 m^2^) was admitted to our hospital because of worsening angina (CCS class 4) and congestive heart failure (NYHA class III). His coronary angiogram showed a left dominance system with a severe 90% stenosis of the left anterior descending artery (LAD), 80% of the first diagonal branch, and 80% of the posterior descending artery (PDA). His LV function was depressed to 30% by left ventriculography and to 35%–40% by echocardiography. Therefore, the patient was referred for CABG surgery and consent to participate in the IMPACT-CABG study. As requested by protocol, a preoperative stress echocardiography and magnetic resonance imaging (MRI) were done and they showed apical necrosis with aneurysm formation, hypokinesia of mid and basal regions of the anteroseptal and anterolateral segments, and akinesia of the inferoapical segment. On the morning of the surgery day, the patient underwent BM aspiration from the iliac crest under local anesthesia. Stem cells were prepared in the cell therapy laboratory, and CD133^+^ cells were purified using the CliniMACS CD133 Reagent System from Miltenyi Biotech Inc. On the evening of the same day, the patient underwent CABG surgery and received the left internal thoracic artery on the LAD and a saphenous vein graft on the PDA. Immediately following distal anastomoses, 10 millions autologous CD133^+^ cells were injected directly into the myocardium using a 26 g needle in the anterior and lateral wall of the left ventricle (Figure 1). 

The aortic cross-clamp time and the total cardiopulmonary bypass (CPB) time were 29 minutes and 45 minutes, respectively. The perioperative course was uneventful without any in-hospital complication related to neither the research protocol nor the surgery. The patient was discharged from the hospital after 7 days. At 6-month followup, the patient symptoms improved to NHYA class I, he was free from angina and his LV ejection fraction was dramatically increased to 60% as assessed by echocardiography (Table 1). 

The regional motion also improved: contractility of the apical region enhanced significantly and the left anteroseptal segments were only slightly hypokinetic. The MRI study demonstrated a spectacular improvement of the perfusion in all territories with normalization of the ischemia in the anteroapical and inferior territories and with persistent mild ischemia in the antero- and inferoseptal basal segments. Moreover, the LV dilatation was reduced, with smaller volume and an increased myocardial mass. No arrhythmia was detected during the in-hospital or 6-month followup.

## 3. Discussion

Advances in the treatment of MI have led not only to increased survival, but also to multiply the number of patients with heart failure. Endogenous repair mechanisms of the human heart are insufficient for sizeable tissue regeneration, so muscle lost is replaced by noncontractile scar tissue. Stem cell transplantation is now emerging as a valuable therapeutical approach to improve healing of the ischemic heart. Experimental studies have shown that adult BM stem cells are capable of differentiation, regeneration of infarcted myocardium, and induction of myogenesis, as well as promotion of angiogenesis, ultimately leading to a better cardiac contractile performance [1]. Over the past several years, a surge of experimental data from preclinical and clinical studies using cell therapy has emerged providing both a proof of concept and therapeutic promises for post-MI cardiac repair and regeneration.

There is still uncertainty as to which of the stem cell population is most potent in stimulating cardiac repair. Some investigators in recent clinical trials have used unfractionated nucleated BM cells, which contain a mixture of cell populations, resulting in mixed and conflicting results [2]. Experimental studies demonstrated that selected, well-defined hematopoietic stem cells contribute to cardiac repair of the acutely infarcted myocardium by inducing neovascularization and cardiomyogenesis and inhibition of cell death. The hematopoietic stem cells are characterized by the presence of the surface marker CD34. In addition, CD133 has been identified as a marker that is present on the stem cells that coexpress not only CD34 but also other markers such as c-kit. It may be hypothesized that the use of these CD133^+^ cells may involve larger or more primitive group of stem cells than selection based only on the use of CD34^+^ marker. The use of nonhomogenous or nonselected stem cells precursors may contribute to the regeneration of necrotic myocardium and blood vessels but does not allow for the characterization of optimal cell type for cardiac repair. Moreover, different cell types may compete for the engraftment in the injured myocardium. It has been shown that selected BMSC displayed a 7-fold higher retention in the infarcted myocardium, when compared to unfractionated and unselected BM stem cells [3]. Indeed, experimental studies demonstrated that CD133^+^-selected well-defined hematopoietic stem cells contribute to cardiac repair of the acutely infarcted myocardium by inducing neovascularization, inhibition of apoptosis and cardiomyogenesis [4]. They possess high engraftment, pluripotent, and angiogenic capacities and appear to be valuable for cardiac repair in experimental MI [5]. In a phase I pilot study, Bartunek et al. demonstrated the feasibility, safety, and functional effects of intracoronary administration of selected autologous CD133^+^ BM progenitors in patients with recent MI [6]. 

We initiated in 2007 the COMPARE-AMI trial, the first Canadian experience with autologous CD133^+^ stem cells transplantation for the treatment of acute ischemic cardiomyopathy. This double-blind randomized controlled trial is investigating the feasibility, safety, and efficacy of intracoronary administration of these selected BM stem cells compared to placebo in patients with LV dysfunction following stented acute MI [7]. To date, 30 patients were successfully enrolled and the one-year safety was recently reported with no protocol-related complication such as death, MI, stroke, or sustained ventricular arrhythmia [8]. 

Patients with chronic ischemic cardiomyopathy referred for CABG may present reduced cardiac function due to previous MI; therefore, we hypothesized that IM injection of CD133^+^ stem cells at the time of surgery could improve the repair of the injured myocardium. In a clinical trial, IMPACT-CABG: IMPlantation of Autologous CD133^+^ sTem cells in patients undergoing CABG, we set out to assess the safety, feasibility, and efficacy of IM delivery of CD133^+^ stem cells compared to placebo at the time of CABG surgery in patients with chronic ischemic cardiomyopathy (for details, http://clinicaltrials.gov, study no. NCT01033617). Adult patients ≤75 years of age referred for CABG are eligible if they have reduced LV systolic function (LV ejection fraction ≤45% but ≥25%) evaluated by echocardiography or left ventriculography. The BM aspiration is performed from the posterior iliac crest under local anesthesia using the standard technique to obtain a total volume of 100 mL. Enriched CD133^+^ stem cells are selected using the CliniMACS CD133^+^ Reagent System (Miltenyi Biotech) which utilizes super-paramagnetic particles composed of iron oxide conjugated to antibodies. The cells are efficiently separated using a high-gradient magnetic separation column. The placebo solution is prepared in a way that is indistinguishable from the cells suspension. Purity is evaluated at 90%, and the amount of cells injected is around 10 × 10^6^ cells. On the same day of cell preparation, patients undergo conventional CABG surgery with midline sternotomy and CPB. Following the CABG procedure, prior to weaning from CPB, the suspension containing CD133^+^ cells or placebo (2.0 mL) is injected into the infarct border zone of myocardium identified as being akinetic or severely hypokinetic. Direct IM injections are made using multiple (approximately 10–15) 0.2 mL aliquots of suspension. The first five patients are treated in an open-labeled fashion with CD133^+^ stem cells to demonstrate the feasibility and safety of the protocol. Subsequently, additional patients will be randomized in a double-blinded design. Patient data will be unblinded at 6 months for end-point assessment, but patients will be followed for a total of 18 months from surgery to assess long-term safety. This pilot study will support future larger and multicentric studies.

In summary, we report the 6-month followup of the first IMPACT-CABG patient. This 59-year-old male with several comorbidities presented congestive heart failure and severe LV dysfunction. However, both stress echocardiography and MRI demonstrated viability in the anterolateral wall with necrosis of the inferior and apical segments of the left ventricle. The patient underwent CABG followed by IM CD133^+^ cells injection in the anterior and lateral wall of the left ventricle. There was no protocol-related complication. At 6 months, the patient showed a significant clinical improvement. In addition, his echocardiography demonstrated a considerable amelioration in cardiac function with a reduction in wall motion score index and his MRI showed a significant improvement in perfusion and viability of the injected area. 

 Although several other studies are examining different cell populations, we believe CD133^+^ stem cells to be amongst the most potent cells for myocardial repair. This work represents the first Canadian experience with CD133^+^ stem cells for the treatment of chronic ischemic cardiomyopathy and relies on state-of-the-art methods for assessing myocardial functional recovery and viability. The remarkable and encouraging results from the first treated patient support the continuation of IMPACT-CABG trial, and by randomization between CABG combined to stem cell versus CABG combined to placebo, this trial will prove the safety of the procedure and possibly the beneficial effects of the cellular therapy. This novel therapy may become an important therapeutic adjunct to conventional treatment for coronary artery diseases.

## Figures and Tables

**Table 1:** 

	Baseline	6 months post CD133^+^
Echocardiography		
LVEF bi plan %	41	60
Wall motion score (WMS)	37	22
Wall motion score index (WMSI)	2.3	1.4

MRI		
LVEDV mL (mL/m^2^)	179 (107)	178 (106)
LVESV mL (mL/m^2^)	111 (66)	94 (56)
LVEF %	38	48
LV mass gr (gr/m^2^)	118 (70)	140 (83)
Stroke volume mL	68	84

LVEF: left ventricular ejection fraction; LVEDV: left ventricular end-diastolic volume; LVES: left ventricular end systolic volume; MRI: magnetic resonance imaging.
